# Molluscicidal and Cercaricidal Effects of *Myrciaria floribunda* Essential Oil Nanoemulsion

**DOI:** 10.3390/molecules28165944

**Published:** 2023-08-08

**Authors:** Francisco Paiva Machado, Diogo Folly, Ricardo Esteves, Bettina Monika Ruppelt, Victoria Marques da Silva, Ana Paula dos Santos Matos, José Augusto Albuquerque dos Santos, Leonardo da Silva Rangel, Marcelo Guerra Santos, Natalia Lidmar von Ranke, Carlos Rangel Rodrigues, Eduardo Ricci-Junior, Leandro Rocha, Robson Xavier Faria

**Affiliations:** 1 Laboratório de Tecnologia de Produtos Naturais (LTPN), Departamento de Tecnologia Farmacêutica, Faculdade de Farmácia, Universidade Federal Fluminense, Rua, Mario Viana, 523, Santa Rosa, Niterói 24241-000, RJ, Brazil; 2 Programa de Pós-Graduação em Biotecnologia Vegetal e Bioprocessos (PBV), Universidade Federal do Rio de Janeiro, Rio de Janeiro 21941-599, RJ, Brazil; 3 Laboratório de Desenvolvimento Galênico (LADEG), Departamento de Fármacos e Medicamentos, Faculdade de Farmácia, Universidade Federal do Rio de Janeiro, Rio de Janeiro 21941-599, RJ, Brazil; 4 Laboratório de Avaliação e Promoção da Saúde Ambiental (LAPSA), Instituto Oswaldo Cruz, Rio de Janeiro 21040-360, RJ, Brazil; 5 Programa de Pós-Graduação em Ciências e Biotecnologia (PPBI), Universidade Federal Fluminense, Niterói 24241-000, RJ, Brazil; 6 Departamento de Ciências, Faculdade de Formação de Professores, Universidade do Estado do Rio de Janeiro, Dr. Francisco Portela 1470, São Gonçalo 24435-000, RJ, Brazil; 7 Laboratório de Modelagem Molecular e QSAR (ModMolQSAR), Faculdade de Farmácia, Universidade Federal do Rio de Janeiro, Rio de Janeiro 21941-599, RJ, Brazil

**Keywords:** Myrtaceae, pesticides, essential oil, schistosomiasis control, nanodispersion, natural products

## Abstract

Schistosomiasis is a tropical disease transmitted in an aqueous environment by cercariae from the *Schistosoma* genus. This disease affects 200 million people living in risk areas around the world. The control of schistosomiasis is realized by chemotherapy, wastewater sanitation, health education, and mollusk control using molluscicidal agents. This work evaluates the effects of a nanoemulsion containing essential oil from *Myrciaria floribunda* leaves as a molluscicidal and cercaricidal agent against *Biomphalaria glabrata* mollusks and *Schistosoma mansoni* cercariae. The *Myrciaria floribunda* essential oil from leaves showed nerolidol, β-selinene, 1,8 cineol, and zonarene as major constituents. The formulation study suggested the F3 formulation as the most promising nanoemulsion with polysorbate 20 and sorbitan monooleate 80 (4:1) with 5% (*w*/*w*) essential oil as it showed a smaller droplet size of approximately 100 nm with a PDI lower than 0.3 and prominent bluish reflection. Furthermore, this nanoemulsion showed stability after 200 days under refrigeration. The *Myrciaria floribunda* nanoemulsion showed LC_50_ values of 48.11 µg/mL, 29.66 µg/mL, and 47.02 µg/mL in *Biomphalaria glabrata* embryos, juveniles, and adult mollusks, respectively, after 48 h and 83.88 µg/mL for *Schistosoma mansoni* cercariae after 2 h. In addition, a survival of 80% was observed in *Danio rerio,* and the in silico toxicity assay showed lower overall human toxicity potential to the major compounds in the essential oil compared to the reference molluscicide niclosamide. These results suggest that the nanoemulsion of *Myrciaria floribunda* leaves may be a promising alternative for schistosomiasis control.

## 1. Introduction

Schistosomiasis is a disease transmitted by parasites from the *Schistosoma* genus. The main species responsible for the disease are *Schistosoma hematobium*, *Schistosoma mansoni*, and *Schistosoma japonicum* [1]. The life cycle of the *S. mansoni* parasite is divided between two hosts: the first cycle occurs in aqueous media in mollusks of the genus *Biomphalaria*, especially *Biomphalaria glabrata*, and the second and definitive cycle occurs in humans [2]. In the environment, the parasite eggs hatch and liberate the first larval stage miracidia, which invade the mollusks of the *Biomphalaria* genus. After a few weeks, the trematode matures into the second larval stage, which is responsible for human infection, and leaves the mollusk as cercariae [2,3]. In humans, cercariae travel through the blood vessels until they reach the liver and become mature worms. Then, *Schistosoma* sp. realizes the sexual cycle and lays its eggs into the mesenteric vessels, being excreted in the feces [4]. Without sanitation, excreta can contaminate the aquatic environment again and restart the cycle [1,2,4].

The principal areas affected by schistosomiasis are tropical and subtropical regions that are mainly poor communities with low or no access to drinking water and basic sanitation [2]. This disease affects over 290 million people around the world living in endemic areas [3]. Strategies for the prevention and control of bilharzia are based on treating the population living in risk areas, improving basic sanitation, providing access to safe drinking water, hygiene education, chemotherapy, and snail control [3]. Niclosamide is the molluscicide agent recommended to control *B. glabrata* mollusks [2]. However, it is toxic to aquatic species and undergoes photodegradation [2,5]. Furthermore, niclosamide frequently needs reapplication [5]. Therefore, developing new products as an alternative to niclosamide is necessary. For this reason, research into novel products of natural origin represents an interesting alternative to obtaining a biorational molluscicide with a less negative impact on public and environmental health [1,5,6,7,8].

Nanostructured systems are in the spotlight for developing novel pesticide alternatives [9,10]. Nanoemulsions (NEs) are thermodynamically unstable colloidal dispersions of two immiscible liquids containing nanometer-sized droplets between 20 and 200 nm [11,12]. Nanoemulsification is a strategy that has been adopted for the application of essential oils or lipophilic extracts in aqueous media because of their hydrophobicity [13,14,15]. Volatile oils are added to surfactants to form droplets and prevent the separation of the oil and aqueous phases, thus favoring the kinetic stability of the dispersion [16,17]. There are several advantages of nanoemulsions with essential oils, such as the prevention of degradation and volatilization of low molecular weight substances, enabling use in aqueous media, increased bioavailability, and improved bioactivity [18].

*Myrciaria floribunda* (H. West ex Willd.) O. Berg (Myrtaceae) is a native plant from Brazil, popularly known as “Cambui”, “Camboim amarelo”, or “camboim”, and is distributed in sandbank areas next to the coast in the Restinga de Jurubatiba National Park in Rio de Janeiro, Brazil [19]. The fruits are edible and popularly used in northern Rio de Janeiro to flavor alcoholic beverages based on the distilled spirit from sugarcane known as “cachaça” [20]. Previous studies described the pharmacological properties of this essential oil with insecticidal and anticholinesterase effects [21,22,23]. Other authors described the antiproliferative, antioxidant, antifungal, antibacterial, anti-inflammatory, and antinociceptive effects of different extract fractions from this plant [19,24,25,26]. Based on this scenario, this work aims to obtain a novel nanoemulsion with molluscicidal and cercaricidal activity from the essential oil of *Myrciaria floribunda* leaves as an alternative against *Biomphalaria glabrata* and *Schistosoma mansoni* to control schistosomiasis.

## 2. Results and Discussion

**Figure 1:**
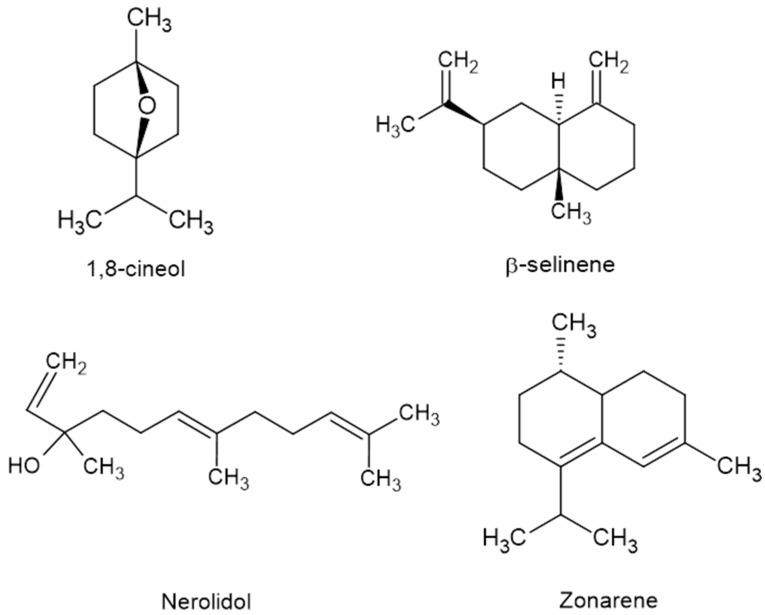
Major components of *Myrciaria floribunda* essential oil from leaves.

The essential oil from *Myrciaria floribunda* yielded 0.9% (*w*/*w*) and showed a clear transparent aspect. The chemical characterization showed 11.33% monoterpene and 80.49% sesquiterpene fractions (Table 1). In addition, 27 substances were identified, and nerolidol (15.4%), β-selinene (13.9%), 1,8-cineole (10.7%), and zonarene (7.67%) were the major components (Figure 1).

The chemical composition of the essential oil of *Myrciaria floribunda* leaves was described by Ramos et al. (2011) [27]. They reported 18.8% monoterpenes and 75.2% sesquiterpenes in the oil. Additionally, the major constituents were nerolidol (32.4%), β-selinene (9.8%), 1,8 cineol (5.8%), and β-terpinene (4.6%) [27]. Another study (Tietbohl et al., 2012) reported 53.9% monoterpenes and 39.6% sesquiterpenes [23]. Additionally, the major components were 1,8 cineole (38.4%), ɣ-himachalene (7%), α-terpineol (5.5%), and zonarene (4.6%) [23]. More recently, Tietbohl et al. (2019) described essential oil with 19.2% monoterpenes and 70% sesquiterpenes and 1,8 cineol (10.4%), β-selinene (8.4%), and α-selinene (7.4%) as major components [22].

The fraction of terpenes in the present study was 11.33% for monoterpenes and 80.5% for sesquiterpenes, which follows the results by Ramos et al. (2010) and Tietbohl et al. (2019) but differs from the terpene fractions described by Tietbohl et al. (2012) [22,23,27]. The major constituents found in this study corroborated the major compounds in the *M. floribunda* essential oil described by Ramos et al. (2010) and Tietbohl et al. (2012; 2019) [22,23,27]. The variations in the chemical compositions of the essential oils were quantitative and qualitative, probably because individual plants suffer different environmental pressures, leading to different adaptations of secondary plant metabolism [28]. Factors such as temperature, collection time, season, extraction, processing, and storage of the raw plant material can lead to variations in the chemical profile of *M. floribunda* essential oil [28].

Essential oils are mainly composed of terpenes and phenylpropanoids, substances of low molecular weight from plant metabolism [28]. They are widely described in the literature as having promising biological properties, generating biotechnological interest in several areas, among them, as molluscicide agents for presenting low cost, high efficiency, biodegradability, and less chemical resistance from parasites or hosts [29]. However, the lipophilic properties of these volatile oils prevent their use for the control of aquatic mollusks such as *Biomphalaria glabrata,* requiring the use of drug delivery systems such as nanoemulsions for their viability to control schistosomiasis [30].

The low-energy by phase-inversion nanoemulsification method was chosen because it does not use organic solvent and avoids the degradation or evaporation of the thermosensitive substances in essential oils [31]. In this work, 11 formulations were prepared with different proportions of surfactants. The criteria to characterize a formulation as a nanoemulsion were an average droplet size <200 nm and PDI <0.3 to be considered a monodisperse system [12,32,33].

**Figure 2:**
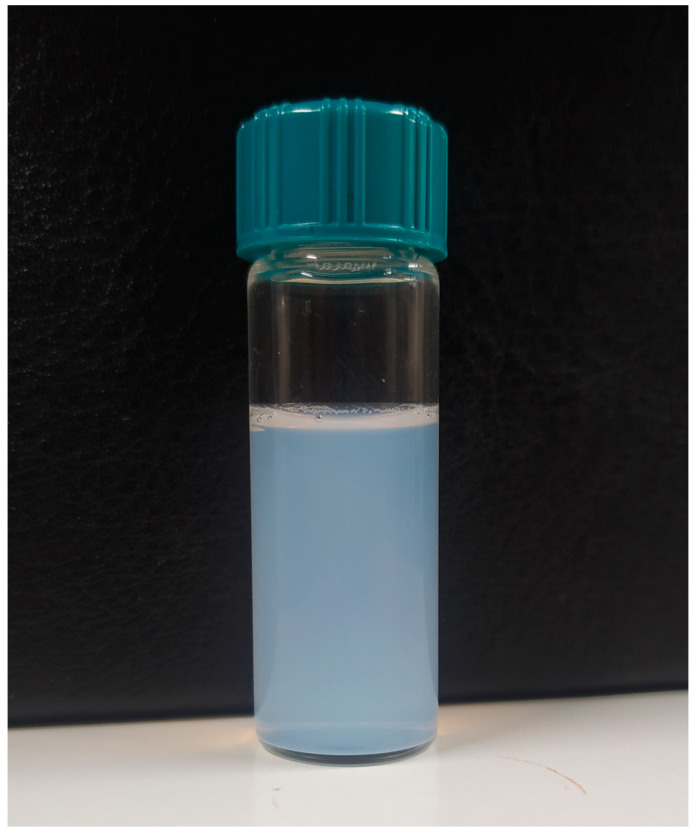
*Myrciaria floribunda* essential oil nanoemulsion (formulation F3) with bluish reflection (dilution 1:40).

**Figure 3:**
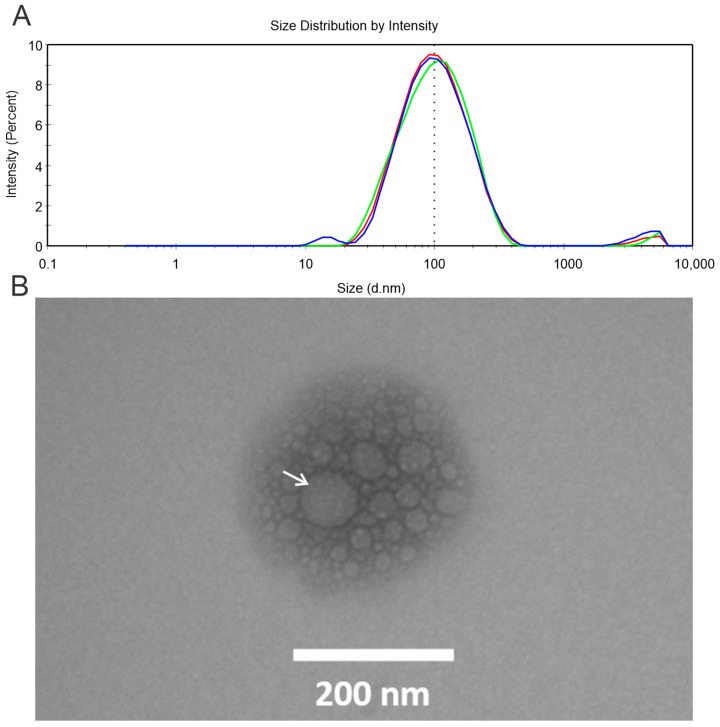
Formulation F3 size distribution by intensity in triplicate (**A**) and transmission electron microscopy (TEM) image showing spherical droplets after 1 h of preparation (**B**).

The formulations with smaller nanodroplets (F1–F3) comprised 4–5% polysorbate 20 and 0–1% sorbitan monooleate (Table 2). Additionally, it showed a bluish-white coloration and an HLB range of 16.70–14.22. To rationalize the experiments, only the F3 formulation (4:1 polysorbate 20 and sorbitan monooleate) was selected to continue the work, owing to its pronounced macroscopic characteristics and smaller droplet size. Also, it showed a typical bluish-white reflection characteristic of light scattering due to the Tyndall effect in nanodispersions (Figure 2) [34]. In addition, the formulation showed a smaller average droplet size of 99.71 ± 0.5220 nm, and the PDI was 0.262 ± 0.013 (Figure 3A). The HLB of 14.22 suggests relatively hydrophilic characteristics of the *M. floribunda* essential oil [17]. The other F5–F9 formulations showed a milky white color, droplet size of >200 nm, and PDI of >0.3. The F10 and F11 formulations (HLB 5.54–4.3) presented visual phase separation and were discarded from the analysis.

Figure 3A shows the size distribution by the intensity of formulation F3, showing the superposition of three measures with an average size of 100 nm and PDI less than 0.3, suggesting a monomodal behavior of nanoemulsion. The TEM image of the F3 formulation after 1 h of preparation at an 89-K magnification (Figure 3B) shows an agglomeration of the droplets due to the sample drying in the copper grid during its preparation and spherical-shaped droplets of approximately 100 nm, which corroborates the mean droplet size observed in the DLS analysis. Smaller droplets are products of the degradation of larger particles, owing to the incidence of the TEM electron beam [32,35].

**Figure 4:**
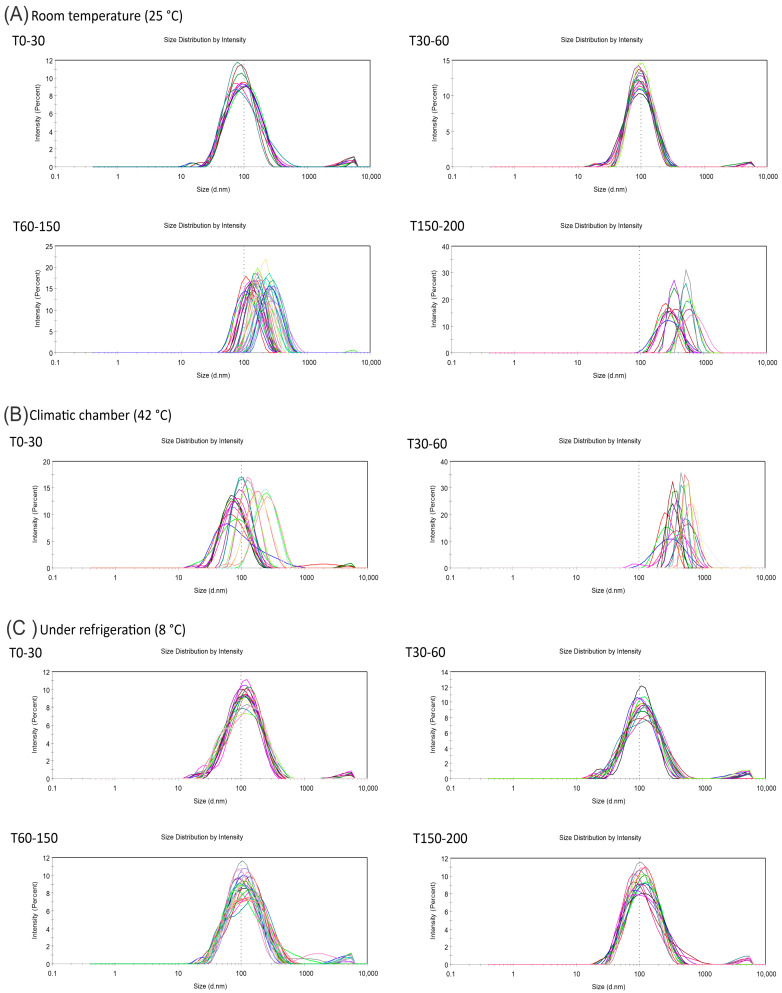
*M. floribunda* nanoemulsion (formulation F3) stability stored at room temperature (**A**), climatic chamber (**B**), and under refrigeration (**C**) over 200 days. Each colored line refers to one day of analysis.

New batches of the F3 formulation were prepared and submitted to the stability study over 200 days (Table 3). The NEs must maintain the mean droplet size and PDI values to be considered stable. The NE stored at room temperature (25 °C) was stable until day 30, presenting a homogeneous particle size and PDI. Then, at 30 to 60 days, the NE decreased the PDI and maintained the size, suggesting the migration of the more hydrophilic compounds of the oil to the aqueous phase. From that point, a gradual increase in the size of the droplets and a decrease in the PDI values were observed, maintaining the characteristic values of the nanoemulsion until day 150, as can be seen in Figure 4A. Afterward, the droplets had a particle size >200 nm, milky white color, and initial phase separation. This can be explained by the fact that more water-soluble oils, such as essential oils, gradually increase the droplet diameter by the Ostwald ripening effect. This mechanism is responsible for the mass transfer from smaller to larger-radius droplets [36]. In this way, it reduces the number of dispersed droplets, presents monomodal behavior, and eventually leads to phase separation, as observed after 200 days in this experiment [36,37].

In the climatic chamber (42 °C), the nanoemulsion showed polymodal behavior and was considered unstable throughout the analysis period (Figure 4B). This formulation presented a milky white color 60 days after preparation. This effect is expected because of the increased energy in the system, leading to the acceleration of the collision of the dispersed droplets, influencing the thermodynamic equilibrium, increasing the solubility of the more hydrophilic constituents, and thus accelerating the Ostwald ripening effect by increasing the coalescence speed, and destabilizing the dispersion [37,38]. However, the formulation stored under refrigeration (8 °C) was stable over time and maintained a bluish-white color, homogeneous particle sizes, and a PDI with the same monomodal behavior overlayed after 200 days of preparation (Figure 4C). This phenomenon was due to the possibility of an increase in viscosity and a decrease in droplet collision in the nanoemulsified system, minimizing coalescence and increasing the viability of the formulation over time [37]. These results suggest that to maintain its physicochemical properties in the long term, the *M. floribunda* NE should preferably be stored and transported under refrigerated conditions.

**Figure 5:**
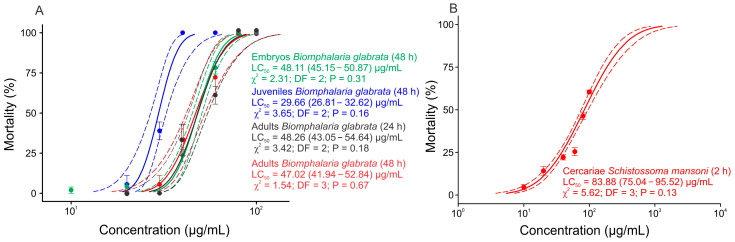
Lethal concentrations of the *Myrciaria floribunda* nanoemulsion (expressed in essential oil) in *Biomphalaria glabrata* embryos, juveniles, and adults (**A**) and *Schistosoma mansoni* cercariae (**B**).

The NE from *M. floribunda* (formulation F3) showed 100% mortality after 24 h at 80 µg/mL, 40 µg/mL, and 80 µg/mL (expressed in essential oil) to *B. glabrata* embryos, juveniles, and adult mollusks, respectively, and 60 µg/mL to *S. mansoni* cercariae after 3 h. The mortality data can be found in the Supplementary Material (Figure S1). The *M. floribunda* NE *B. glabrata* adults (10–12 mm) LC*_50_* after 24 h was 48.26 (43.05–54.64) µg/mL. The LC_50_ after 48 h was 29.66 (26.81–32.62) µg/mL to juvenile mollusks (6–8 mm), 47.02 (41.94–52.84) µg/mL to adults (10–12 mm), 48.11 (45.15–50.87) µg/mL to embryos, and 83.88 (75.04–95.52) µg/mL to cercariae after 2 h (Figure 5). Regarding the lethal effect of the positive control, niclosamide showed 100% cercaria and mollusk mortality (embryos, juvenile, and adults) after 24 h in all groups (*p* < 0.001). At the same time, no mortality was observed in the negative control (distilled water) and NE blank (without essential oil) (*p* < 0.001). The nanoemulsion of *M. floribunda* was effective in all phases of the life cycle of the mollusk *B. glabrata*, showing a more significant effect on juvenile mollusks (6–8 mm). This phenomenon probably occurred because the juvenile organism was not fully developed and was more susceptible to the effects of the nanoemulsion and essential oil components [15].

Several studies for the control of mollusks, especially *B. glabrata* and the parasite *S. mansoni*, have been conducted with products derived from plant origin, generating important information and showing the potential of plants as a source of bioactive metabolites to support the combat of schistosomiasis [5,7,29,39,40,41,42,43]. However, few studies have approached the formulation of these novel natural products for the functional control of aquatic mollusks since the common limitation in these studies is the physicochemical properties of substances, which are often volatile or insoluble in aqueous media and generally require the use of organic solvents such as dimethyl sulfoxide (DMSO) to enable biological testing [29]. In this context, nanoemulsions present themselves as a potential research field to enable these actives in an aqueous medium, especially essential oils. A few authors described similar nanotechnological approaches with nanoemulsions from *Xylopia ochrantha* (Annonaceae) essential oil (24 h/LC_50_ = 50.9 µg/mL), *Ocotea pulchella* (Lauraceae) essential oil (24 h/LC_50_ = 45.8 µg/mL), and *Sideroxylon obtusifolium* (Sapotaceae) extract (24 h/LC_50_ = 75.2 µg/mL) against *B. glabrata* mollusks, thus corroborating the LC_50_ values found in our study [13,15,44].

According to the World Health Organization, for an essential oil to be considered a promising molluscicide agent, it must have a mortality of at least 90% of the adult mollusk population at concentrations equal to or less than 100 µg/mL [29]. In this context, the nanoemulsion of essential oil from *M. floribunda* leaves can be considered a promising molluscicide formulation with 100% mortality at 80 µg/mL after 24 h and an LC_50_ of 47 µg/mL after 48 h in *B. glabrata* adults.

Evaluation of the environmental and human toxicity of molluscicides is crucial to ensure the safety and future adhesion of the product since the human population, as well as non-target organisms, may consume hydric resources from polluted water bodies close to urban or peri-urban environments in endemic regions of tropical diseases, including schistosomiases [1,29]. For this reason, it is important to develop new selective products to avoid harmful effects on the environment or human health [29]. In this study, *Danio rerio* (Zebrafish) was used as an aquatic non-target organism. The Organization for Economic Co-operation and Development (OECD) indicates it as an animal model for toxicological and ecotoxicological studies [45,46]. The *M. floribunda* NE, besides presenting promissory molluscicidal activity in the broad spectrum of the *B. glabrata* lifecycle, has not shown significant toxicity to Zebrafish. The applied oral dose corresponded to 50 µg/mL (expressed in essential oil), approximately the LC_50_ against the target organism *Biomphalaria glabrata*. In the first 6 h, 10% of lethality was observed; then, another 10% of lethality occurred at 48 h. There was an 80% in the survival of the adult *Danio rerio*. No changes related to equilibrium, swimming behavior, ventilatory function, skin pigmentation, visible abnormalities, or weight were observed after 48 h of exposure, suggesting low toxicity to the NE with *M. floribunda* essential oil [45].

The in silico ecotoxicity results are shown in Table 4. The major compounds of the oil were analyzed individually in comparison with the reference molluscicide niclosamide. Regarding the bioconcentration factor (BCF), all the analyzed compounds presented higher values than niclosamide. From the compounds analyzed, 1,8-cineole was predicted to have a moderate accumulation in aquatic organisms. On the other hand, β-selinene and zonarene were predicted to have a very high BCF. Therefore, these substances are expected to accumulate in lipophilic tissues. Nerolidol was the only terpenoid evaluated as supposedly biodegradable.

Regarding the aquatic toxicity of *Tetrahymena pyriformis* species, all substances were predicted to be more toxic than niclosamide. Regarding *Daphnia magna*, all the analyzed compounds tended to be less toxic than niclosamide, especially 1,8-cineole. Finally, for the *Pimephales promelas* species, all compounds analyzed were predicted to be more toxic than niclosamide, except for 1,8-cineole, which was predicted to have considerably low toxicity for this species. The endocrine toxicological results indicated that niclosamide, β-selinene, and nerolidol could interact with the androgen receptor. This result suggests a low endocrine toxicological event of these compounds.

Most substances from the essential oil of *M. floribunda* leaves showed a lower overall human toxicity potential than niclosamide. Concerning environmental toxicity, only 1,8-cineole exhibited better parameters than niclosamide. However, it must be said that the *M. floribunda* essential oil is a phytocomplex; in other words, it presents different proportions of terpenoids in its composition and, in this context, may reduce the potentially toxic effects of the essential oil in relation to niclosamide.

## 3. Materials and Methods

### 3.1. Plant Material

The collection of the *Myrciaria floribunda* leaves was realized at the Restinga de Jurubatiba National Park, Rio de Janeiro, Brazil (22°12′98.6″ S–41°35′00.7″ O, 22°12′99.8″ S–41°35′01.8″ O). The collection and research of the vegetal material were authorized by the Chico Mendes Institute of Biodiversity Conservation (ICMbio/Brazil) under n° 13659-14 and SisGen code A314288. An *M. floribunda* voucher specimen was deposited at the Herbarium of the Faculdade de Formação de Professores (FFP) under registration RFFP: 13.789 of the Universidade do Estado do Rio de Janeiro (UERJ).

### 3.2. Essential Oil Extraction

Fresh leaves (3120 g) of *Myrciaria floribunda* were separated from the stem and crushed in distilled water. Then, they were conditioned in a round-bottom flask and hydrodistilled in a modified Clevenger for 4 h to extract the essential oil. Finally, the essential oil was filtered in anhydrous sodium sulfate (Na_2_SO_4_), collected in an amber glass vial, and stored in a freezer (−20 °C).

### 3.3. Essential Oil Characterization

The *M. floribunda* essential oil (1 µL) was solubilized in dichloromethane (GC grade) at proportion 1:100 mg/μL, then characterized by a gas chromatograph model QP2010 (Shimadzu) coupled with a mass spectrometer (MS) and a gas chromatograph model GC-2014 (Shimadzu) equipped with a flame ionization detector (FID).

The gas chromatographic conditions were the following: The column was an RTX-5 column (0.25 mm ID, 30 m in length, 0.25 µm film thickness). The carrier gas was helium, with a flow rate of 1 mL/min. The temperature of the injector was 260 °C with split injection (ratio 1:40). The temperature in the oven started at 60 °C and then increased to 290 °C at a rate of 3 °C/min. The mass spectrometry conditions included electron ionization of 70 eV and a scan rate of 1 scan/s.

The arithmetic index was calculated with the retention time intervals of a standard mixture of aliphatic hydrocarbons (C7–C40) analyzed under the same gas chromatographic conditions described above. The essential oil components were identified by comparing their retention indices, arithmetic indices, and mass spectra with spectral databases (Adams, 2017). The compound’s MS fragmentation pattern was analyzed in relation to the suggestion from the NIST GC mass spectrum libraries. The quantification of the terpenoids was realized using flame ionization gas chromatography (GC-FID) under the same GC–MS conditions except for the FID temperature (290 °C). The percentages of the substances in the essential oil were obtained by the FID peak area normalization method.

### 3.4. Nanoemulsion Preparation and Characterization

The formulations were prepared by the low-energy phase-inversion method with modifications [45]. To prepare the formulations and determine the HLB value, 11 mixtures with different ratios of nonionic surfactants polysorbate 20 (HLB 16.7) and sorbitan monooleate 80 (HLB 4.3) were prepared (Table 5). The nonionic surfactants were added to the essential oil (5% *w*/*w*) and homogenized by magnetic stirring at 500 rpm for 30 min at room temperature (25 °C). Then, the aqueous phase was slowly dripped onto the oil phase under the same conditions for 60 min. The parameters for the characterization were average droplet size (in nm) and polydispersity index (PDI), analyzed by the dynamic light scattering (DLS) in a Zetasizer Nano, model S90 device (Malvern Panalytical, UK). The measurements were performed with the samples at room temperature (25 °C) in a 1:50 dilution in distilled water. The required HLB for the *M. floribunda* essential oil was calculated with Equation (1) [46].

(1) $$HLBr=\frac{\left(HLBa\times A\%+HLBb\times B\%\right)}{100}$$

where the required *HLBr* is the resulting value of two surfactants mixture, *HLBa* is the more hydrophobic surfactant, *A*% is the percentage of the hydrophobic surfactant, *HLBb* is the hydrophilic surfactant, *B*% is the percentage of the hydrophobic surfactant, and *A*% + *B*% = 100.

### 3.5. Stability Study

The promising NE formulation was selected to carry out the stability study for 200 days. The NEs were prepared and stored under different thermal conditions: room temperature (25 ± 2 °C), refrigeration (8 ± 2 °C), and in a climatic chamber (42 ± 2 °C) [47]. The mean droplet size (in nm) and polydispersity index (PDI) were evaluated with NE at room temperature (25 °C) several times (0, 7, 15, 30, 60, 90, 120, 150, and 200 days) after preparation under the three storage conditions.

### 3.6. Transmission Electron Microscopy (TEM)

The morphology of the NE with promising DLS values after 1 h of preparation was characterized by transmission electron microscopy (TEM) with a Morgagni 268/FEI (FEI Company, Eindhoven, The Netherlands). First, the NE was diluted in a proportion of 1:1 in distilled water. Then, 5 µL was conditioned in a copper grid coated with formvar, dried in a desiccator for 60 min, and analyzed.

### 3.7. Ovicidal Assay

The ovicidal capacity of the nanoemulsion was tested on embryos of *B. glabrata* at 72 h of life. For this, in triplicate, following Araújo et al. (2019) methodology and using 24-well plates, embryos (*n* = 150) were placed in a well and exposed to 2 mL of the nanoemulsion formulation. Embryos were exposed for 48 h to the following NE concentrations: 100, 80, 60, 40, 20, and 10 µg/mL (expressed in essential oil). Mortality was evaluated continuously after 24 h and 48 h. The negative control was distilled water, and niclosamide (2 µg/mL) was used as the positive control. Additionally, the blank NE (without active oil) was evaluated.

### 3.8. Molluscicidal Assays

The mollusks were collected at Sumidouro (RJ, Brazil) and kept in breeding tanks at the Lauro Travassos Pavilion of the Oswaldo Cruz Institute (Rio de Janeiro). They were maintained in chlorine-free water and fed with *Lactuca sativa* L. (1758). For the molluscicidal assays (*n* = 108), 24-well plates containing 2 mL of NE were used in each well at the following concentrations: 10, 20, 40, 60, 80, and 100 µg/mL (expressed in essential oil). Next, *B. glabrata* juvenile (6–8 mm) and adult (10–12 mm) mollusks were individually placed in the wells [48]. The same volume was used for the negative control (distilled water), blank NE (without essential oil), and positive control (niclosamide at 2 µg/mL). Mortality was assessed at 24 h and 48 h. The mortality criteria were the release of hemolymph, absence of retraction, and exaggerated retraction into the shell.

### 3.9. Cercaricidal Assay

The *M. floribunda* NE was also evaluated against the cercariae of the parasite *S. mansoni* (*n* = 480). For this purpose, in triplicate, the mortality of cercariae in suspension exposed to NE was evaluated in a 24-well plate, totaling 2 mL per test well. Each group (*n* = 80) was exposed to NE at 100, 80, 60, 40, 20, and 10 µg/mL (expressed in essential oil) for 4 h. The mortality was evaluated after every hour for 4 h after nanoemulsion exposure. The negative control was distilled water and the blank NE (without active oil) at 100 µg/mL, and the positive control was niclosamide (2 µg/mL).

### 3.10. In Silico Environmental Toxicity Analysis

ADMET Predictor™ (version 9.5; Simulations Plus, Lancaster, CA, USA) was used to evaluate the reference molluscicide niclosamide and the essential oil terpenoids molecular structure and experimental data to create the QSAR models to predict the biological properties of the essential oil major compounds. The endpoints were bioconcentration, biodegradation, aquatic toxicity at different trophic levels (*Tetrahymena pyriformis*, *Daphnia* (water flea), and *Pimephales promelas*), endocrine toxicity (estrogenic and androgenic hormones) and toxicological risk.

### 3.11. Acute Oral Toxicity in Non-Target Danio rerio

The experiment followed the ARRIVE guidelines of animal welfare regulation and was approved by the Ethics Committee of Instituto Vital Brazil protocol number 003/2019 [49]. Male *Danio rerio* (Zebrafish) weighing 400–450 mg, provided by the Alternative Methods to Animal Use Laboratory of the Vital Brazil Institute, were kept in a rack with the following water control parameters: pH = 7.0 ± 1, a temperature of 26 ± 2 °C, ammonia of 0 ppm, and a photoperiod (light/dark) of 12 h/12 h.

Ten *Danio rerio* were randomly distributed in equal numbers in five experimental tanks (15 × 8 × 12 cm^3^) containing 1 L of water. The dose corresponding to 50 µg/mL (expressed in essential oil) of the *M. floribunda* nanoemulsion was administered orally (gavage). The clinical signs related to balance, swimming behavior, ventilatory function, skin pigmentation, and visible abnormality were observed at 0, 3, 6, 24, and 48 h after oral administration. The mortality criteria were no visible reaction after touching the caudal peduncle. The animals were weighed before and after the experiment [50]. The evaluation and use of clinical signs for humanized endpoint assessment were observed [51]. In the end, the animals were euthanized using a eugenol solution [52].

### 3.12. Statistical Analysis

The LC_50_ estimations were realized through probit analysis with SAS software [53], and survival and one-way analysis of variance (ANOVA) followed by Tukey’s post-test was performed using GraphPad Prism (ver. 8) with a significance level of *p* < 0.001.

## 4. Conclusions

This study revealed a stable biotechnological nanoemulsion containing *Myrciaria floribunda* (Myrtaceae) essential oil as a potential alternative to control *Biomphalaria glabrata* mollusks, the intermediate host of schistosomiasis, presenting promising mortality effects in a broad spectrum of the mollusk life cycle and cercariaes, the human infective form of *Schistosoma mansoni*.

## 5. Patents

This work resulted in an invention patent deposited at the Brazilian National Institute of Industrial Property (INPI) process number BR 10 2020 026179 7.

## Figures and Tables

**Table 1:** Chemical characterization of the essential oil from leaves of *Myrciaria floribunda* GC–MS and GC-FID.

	Retention Index	Arithmetic Index	Arithmetic Index Calculated	Substances	%
1	8.321	1023	1022	O-Cymene	0.19
2	8.459	1027	1026	1,8-Cineole	10.70
3	14.963	1194	1186	α-Terpineol	0.44
4	22.355	1369	1373	α-Ylangene	3.43
5	24.126	1412	1417	β-Caryophylenne	2.28
6	24.920	1431	1439	Aromadendrene	0.27
7	25.581	1447	1452	α-Humulene	1.15
8	26.333	1466	1476	β-Chamigrene	1.87
9	26.937	1481	1489	β-Selinene	13.28
10	27.235	1488	1498	α-Selinene	6.13
11	27.387	1492	1500	α-Muurolene	0.49
12	27.493	1495	1509	α-Bulnesene	0.45
13	28.136	1511	1511	δ-Amorphene	4.80
14	28.292	1515	1521	trans-Calamenene	0.43
15	28.819	1529	1528	Zonarene	7.69
16	29.010	1534	1545	Selina-3,7(11)-diene	6.54
17	29.654	1550	1559	Germacrene B	0.50
18	29.886	1556	1561	Nerolidol	15.43
19	30.560	1574	1582	Caryophyllene oxide	0.76
20	31.176	1590	1595	Cubeban-11-ol	0.29
21	32.506	1625	1622	10-epi-γ-Eudesmol	0.66
22	33.311	1647	1649	β-Eudesmol	1.24
23	33.439	1651	1658	neo-Intermedeol	4.41
24	33.836	1661	1665	Intermedeol	1.56
25	34.892	1690	1700	Eudesm-7(11)-en-4-ol	1.48
26	35.702	1713	1714	Farnesol	2.05
27	39.756	1829	1821	(2Z,6E)-Farnesyl acetate	3.33
	Total identified				91.82
	Monoterpene hydrocarbons				0.19
	Oxygenated monoterpenes				11.14
	Monoterpenes: total				11.33
	Sesquiterpenes hydrocarbons				49.31
	Oxygenated sesquiterpenes				31.18
	Sesquiterpenes: total				80.49

**Table 2:** Average droplet size, polydispersity index (PDI), and required hydrophilic-lipophilic balance (HLB) values of formulations F1–F9 of *Myrciaria floribunda* essential oil.

Formulation	Droplet Size (nm)	Polydispersity Index	Hydrophilic-Lipophilic Balance
F1	171.8 ± 1.3	0.271 ± 0.011	16.7
F2	120.5 ± 0.92	0.249 ± 0.009	15.46
F3	99.71 ± 0.52	0.262 ± 0.013	14.22
F4	196.4 ± 1.4	0.322 ± 0.105	12.98
F5	311.5 ± 12.80	0.204 ± 0.134	11.74
F6	592.8 ± 84.40	0.672 ± 0.340	10.5
F7	1532 ± 646.25	0.520 ± 0.481	9.26
F8	1750 ± 817.41	0.439 ± 0.430	8.02
F9	1461 ± 263.65	1.0 ± 0	6.78

**Table 3:** Stability study of *Myrciaria floribunda* nanoemulsion (formulation F3) at different temperatures.

	25 °C		8 °C		42 °C	
	Average Size (nm)	Polydispersity Index	Average Size (nm)	Polydispersity Index	Average Size (nm)	Polydispersity Index
T00	87.2 ± 1.363	0.267 ± 0.010	96.6 ± 0.9917	0.263 ± 0.009	69.6 ± 1.767	0.283 ± 0.013
T07	84.4 ± 1.05	0.268 ± 0.008	98.1 ± 0.615	0.261 ± 0.003	72.0 ± 0.87	0.186 ± 0.010
T15	84.5 ± 1.517	0.261 ± 0.010	98.2 ± 1.103	0.248 ± 0.0	91.9 ± 0.2553	0.112 ± 0.021
T30	80.4 ± 0.9721	0.241 ± 0.008	94.3 ± 0.6274	0.268 ± 0.007	161.2 ± 2.237	0.148 ± 0.017
T60	91.4 ± 1.231	0.178 ± 0.008	97.8 ± 1.250	0.273 ± 0.009	533.8 ± 32.51	0.151 ± 0.080
T90	130 ± 0.8505	0.098 ± 0.008	100.4 ± 1.195	0.271 ± 0.004	843.5 ± 67.30	0.417 ± 0.519
T120	188.5 ± 1.801	0.141 ± 0.052	101.8 ± 1.041	0.285 ± 0.019	2603 ± 82.56	0.243 ± 0.031
T150	276.3 ± 5.408	0.126 ± 0.029	97.0 ± 0.6504	0.265 ± 0.013	2988 ± 423	0.676 ± 0.208
T200	580.0 ± 20.46	0.165 ± 0.105	102.4 ± 1.386	0.219 ± 0.008	2998 ± 114	0.370 ± 0.143

**Table 4:** *Myrciaria floribunda* essential oil major compound ecotoxicity results.

Compounds	Bioconcentration Factor	Biodegradation	Aquatic Toxicity			Endocrine Receptor Binding		TOX-Risk *
			*Tetrahymena* pIGC_50_ *	*Daphnia* LC_50_ *	Minnow LC_50_ *	Androgen Receptor *	Estrogen Receptor *	
Niclosamide	6.65	No	1.968	1.752	3.612	Toxic	Nontoxic	2
Zonarene	1497.046	No	1.003	11.855	0.421	Nontoxic	Nontoxic	1
β-selinene	1951.705	No	1.287	2.110	0.369	Toxic	Nontoxic	-
1,8-Cineole	61.434	No	0.041	227.511	149.762	Nontoxic	Nontoxic	0
Nerolidol	566.945	Yes	0.774	2.863	1.531	Toxic	Nontoxic	1

* Th_pyr_pIGC50: median inhibition of *Tetrahymena pyriformis* after 40 h of exposure; Daphnia_LC50: median lethal concentration (mg/L) *Daphina. magna* population; Minnow_LC50: median lethal concentration (mg/L) of minnows. Andro_Filter and Estro_Filter: evaluators of a substance’s affinity in binding to the androgen/estrogen receptor. TOX-Risk: potential liabilities of a substance.

**Table 5:** *Myrciaria floribunda* formulation (F1–F11) compositions.

	Oil Phase % (*w*/*w*)	Aqueous Phase % (*w*/*w*)	Essential Oil % (*w*/*w*)	Polysorbate 20% (*w*/*w*)	Sorbitan Monooleate 80% (*w*/*w*)
F1	10.0	90.0	5.0	5.0	0.0
F2	10.0	90.0	5.0	4.5	0.5
F3	10.0	90.0	5.0	4.0	1.0
F4	10.0	90.0	5.0	3.5	1.5
F5	10.0	90.0	5.0	3.0	2.0
F6	10.0	90.0	5.0	2.5	2.5
F7	10.0	90.0	5.0	2.0	3.0
F8	10.0	90.0	5.0	1.5	3.5
F9	10.0	90.0	5.0	1.0	4.0
F10	10.0	90.0	5.0	0.5	4.5
F11	10.0	90.0	5.0	0.0	5.0

## Data Availability

The samples of essential oil are available from the authors.

## Supplementary Materials

The following supporting information can be downloaded at: https://www.mdpi.com/article/10.3390/molecules28165944/s1, Figure S1.
