# Supplementary figures and images for: Molluscicidal and Cercaricidal Effects of Myrciaria floribunda Essential Oil Nanoemulsion
Source: Molecules. 2023 Aug 8;28(16):5944. doi: 10.3390/molecules28165944 (PMC10458193; doi:10.3390/molecules28165944)

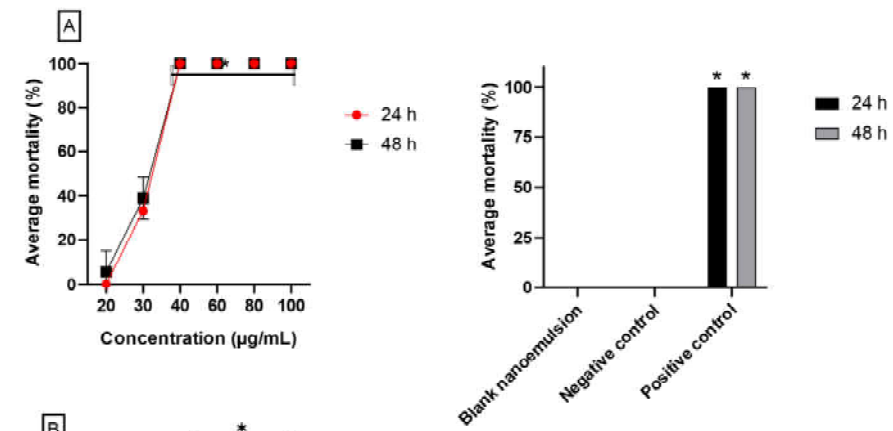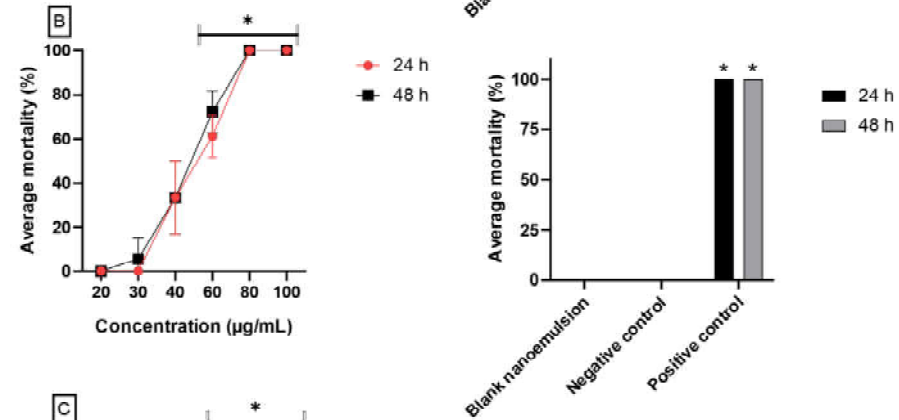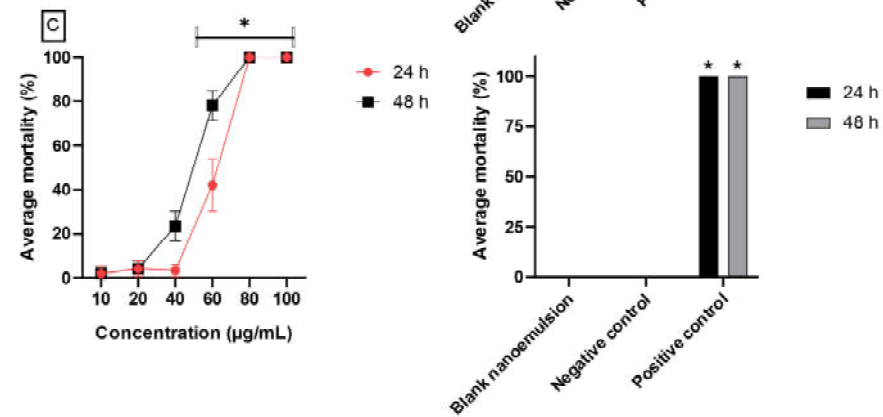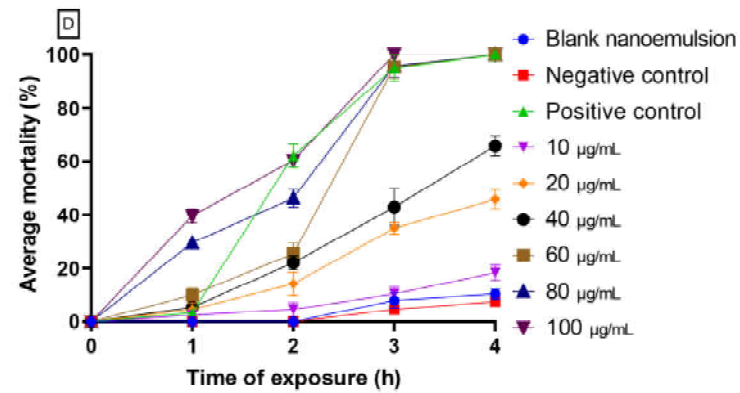

Supplement: Supplementary file 1 [file molecules-28-05944-s001.zip › molecules-2130340-supplementary.pdf]
